# Cooking the Books

**Published:** 1991-09

**Authors:** Jack Davies


### COOKING THE BOOKS

Mrs. Beeton, after her education in Heidelberg, was probably right. You need both ingredients and the place.

As said before in this column, the kitchen is a dangerous room for matrimonial harmony (well OK, I must confess that we are not yet living in a caravan. Wiser councils prevailed and in any case our Editor intimated that he didn't want contributions from people who are of no fixed address — I think that it is all something to do with correcting proofs. Anyway, Michael Wilson has politely pretended not to hear when pressed on the point).

Back to cooking. Here we do have a definite problem. Straightaway it ought to be pointed out that cooking and the kitchen *chez nous* should provide the classic confirmation of how opposites manage to weather the storms of marriage. My partner is skilled in things culinary, literally a professional, and usually is given to the lighter points of conversation at the end of the day in the kitchen.

I *love* the muddle and mess of cooking. Chinese is my favourite at the moment, and the books on that are tremendous: theory and practice are nicely combined. There are so many methods on offer. Until now I'd never dared try *Feng* or air drying. Next week perhaps. Bamboo steamers, cleavers and woks with lots of smoking oil. Not merely that, but the red peppers, green coriander, yellow bean curd, a heap of wood-ear mushrooms, ginger choppings and garlic piles make it all so festive. Even the pots look fun with the bubbly foam on top of the boiling wheat noodles. It is a pleasure trying to keep the steamed rice and the wok going, whilst at the same time adjusting the oven and putting out the minor incineration on the back left hand gas. Cook's perks in the form of sake or rice wine aren't bad either.

Most kitchens have pots and pans superflous to need. This, I consider a bad mistake. In a team everyone and everything ought to have a go. Maybe you have egg coddlers which haven't been used ever since Aunt Elsie gave them to you on your wedding day. When you adopt this egalitarian approach to cookery, it's really satisfying. Medicine does seem dull when in the full swing of Chinese cooking.

However the proper professional in the house has other views. She points out that it takes time to wash up afterwards. My own personal views about the place of the amateur and the professional in the kitchen usually get very short shrift. It does not seem fair.

In the end, perhaps just reading books about Chinese cookery seems the wiser course. Can anyone lend me a pair of spare ribs just to practice on? Promise, I'll go out into the garage. Come to think of it, that's where we used to keep the barbecue and the camping gas. Who said writing for a journal wasn't a good ideal. I've always wondered what roast suckling pig was like.

First find your garage....

